# Increased resting perfusion of the hippocampus in high positive schizotypy: A pseudocontinuous arterial spin labeling study

**DOI:** 10.1002/hbm.24231

**Published:** 2018-06-08

**Authors:** Gemma Modinos, Alice Egerton, Katrina McMullen, Anna McLaughlin, Veena Kumari, Gareth J. Barker, Steve C. R. Williams, Fernando Zelaya

**Affiliations:** ^1^ Department of Psychosis Studies Institute of Psychiatry, Psychology & Neuroscience, King's College London London United Kingdom; ^2^ Department of Neuroimaging Institute of Psychiatry, Psychology & Neuroscience, King's College London London United Kingdom; ^3^ Department of Psychology Institute of Psychiatry, Psychology & Neuroscience, King's College London London United Kingdom; ^4^ Centre for Cognitive Neuroscience, College of Health and Life Sciences Brunel University London London United Kingdom

**Keywords:** ASL, CBF, medial temporal lobe, MRI, neuroimaging, psychosis proneness, psychosis

## Abstract

Arterial spin labeling (ASL) provides absolute quantification of resting tissue cerebral blood flow (CBF) as an entirely noninvasive approach with good reproducibility. As a result of neurovascular coupling, ASL provides a useful marker of resting neuronal activity. Recent ASL studies in individuals at clinical high risk of psychosis (CHR) have reported increased resting hippocampal perfusion compared with healthy controls. Schizotypy refers to the presence of subclinical psychotic‐like experiences in healthy individuals and represents a robust framework to study neurobiological mechanisms involved in the extended psychosis phenotype while avoiding potentially confounding effects of antipsychotic medications or disease comorbidity. Here we applied pseudo‐continuous ASL to examine differences in resting CBF in 21 subjects with high positive schizotypy (HS) relative to 22 subjects with low positive schizotypy (LS), as determined by the Oxford and Liverpool Inventory of Feelings and Experiences. Based on preclinical evidence that hippocampal hyperactivity leads to increased activity in mesostriatal dopamine projections, CBF in hippocampus, midbrain, and striatum was assessed. Participants with HS showed higher CBF of the right hippocampus compared to those with LS (*p* = .031, family‐wise error corrected). No differences were detected in the striatum or midbrain. The association between increased hippocampal CBF and HS supports the notion that hippocampal hyperactivity might be a central characteristic of the extended psychosis phenotype, while hyperactivity in subcortical dopamine pathways may only emerge at a higher intensity of psychotic experiences.

## 1 INTRODUCTION

Healthy brain function relies on the appropriate regulation of regional cerebral blood flow (CBF) for the delivery of oxygen and glucose to support brain metabolism. Over the past decade, arterial spin labeling (ASL) has emerged as a robust and noninvasive method for acquiring regional CBF maps (Hernandez‐Garcia, Lahiri, & Schollenberger, 2018) with high reproducibility (Gevers et al., 2011; Hermes et al., 2007). In contrast to imaging methodologies that depend on the administration of a contrast agent to measure perfusion, such as dynamic susceptibility contrast (DSC)‐magnetic resonance imaging (MRI), computed tomography (CT) perfusion imaging, single‐photon emission tomography (SPECT), and H_2_[^15^O] positron emission tomography (PET), ASL generates an image by magnetically “labeling” water molecules as an endogenous tracer (Williams, Detre, Leigh, & Koretsky, 1992). The difference between labeled and unlabeled water within the blood reveals parenchymal perfusion, an indirect yet highly correlated measure of neuronal function, in units of milliliters of blood per 100 g of tissue per minute (Alsop et al., 2015). Due to its noninvasive nature and ability to quantitatively measure tissue perfusion, ASL is currently used in routine clinical practice in cerebrovascular disease and neuro‐oncology, and there is a growing interest in its research and clinical applications to psychiatric disease (Grade et al., 2015).

While the anatomy of psychosis involves multiple brain regions and networks, postmortem, preclinical, and clinical imaging studies demonstrate a critical role of the hippocampus in its pathophysiology (Grace, 2010; Heckers, 2001; Tamminga, Stan, & Wagner, 2010). Most studies of resting perfusion in patients with schizophrenia spectrum disorders relative to healthy controls, thus, focused predominantly on the hippocampal region. Overall, these studies reported seemingly mixed results, including increases (Friston, Liddle, Frith, Hirsch, & Frackowiak, 1992; Liddle et al., 1992; Malaspina et al., 2004; Pinkham et al., 2011; Schobel et al., 2013, 2009; Talati et al., 2014; Talati, Rane, Skinner, Gore, & Heckers, 2015), decreases (Kindler et al., 2015; Nordahl et al., 1996; Scheef et al., 2010; Tamminga et al., 1992), or no differences (Horn et al., 2009; Ota et al., 2014; Vita et al., 1995). Beyond the hippocampus, other brain regions of significantly elevated resting perfusion in schizophrenia patients compared with healthy controls have involved the basal ganglia and middle temporal lobes (Pinkham et al., 2011), cerebellum, brainstem, and thalamus (Scheef et al., 2010). Noteworthy, while not directly focusing on the hippocampus as a specific region, initial PET and SPECT studies did not find increased perfusion in medial temporal regions (Andreasen et al., 1997; Catafau et al., 1994; Early, Reiman, Raichle, & Spitznagel, 1987; Parellada et al., 1994). A possible source for the inconsistencies noted among individual studies is that resting perfusion research in patients with schizophrenia is complicated by antipsychotic exposure (Lahti et al., 2006; Lahti, Weiler, Holcomb, Tamminga, & Cropsey, 2009; Medoff, Holcomb, Lahti, & Tamminga, 2001). In this context, recent studies in mostly antipsychotic‐free subjects at clinical high risk of developing psychosis (CHR) compared to healthy controls have found that resting perfusion is increased in hippocampus (Allen et al., 2017, 2016; Schobel et al., 2013), striatum (Allen et al., 2017, 2016; Kindler et al., 2018), and midbrain (Allen et al., 2017, 2016). These findings are consistent with data from preclinical models of psychosis which propose a key role for hippocampal hyperactivity in the emergence of psychotic‐like neurobiological and behavioral phenotypes by disrupting subcortical tonic dopamine neuron firing (Lodge & Grace, 2011). Furthermore, multimodal imaging studies in CHR subjects have demonstrated that hypermetabolism of the hippocampus predicts hippocampal volume loss that is associated with increased glutamate levels (Schobel et al., 2013), and that resting hippocampal perfusion is related to medial prefrontal GABA+ levels in CHR subjects, particularly in those who subsequently develop a psychotic disorder (Modinos et al., 2018). On the basis of the observation of heightened hippocampal perfusion in patients with psychotic disorders (Friston et al., 1992; Liddle et al., 1992; Malaspina et al., 2004; Pinkham et al., 2011; Schobel et al., 2013, 2009; Talati et al., 2014, 2015) and in CHR individuals (Allen et al., 2017, 2016; Schobel et al., 2013), hippocampal hyperperfusion might be a central characteristic of the extended psychosis phenotype.

The continuum model of psychosis posits dimensional continuity between subclinical psychotic‐like experiences in healthy individuals (termed *schizotypy*) and psychotic symptoms in patients with schizophrenia spectrum disorders (Linscott & van Os, 2013; Nelson, Seal, Pantelis, & Phillips, 2013). The high schizotypy (HS) paradigm involves healthy people who are typically non‐help‐seeking and have thus not been exposed to antipsychotic medication. HS individuals can be identified using psychometrically validated self‐report instruments such as the Oxford and Liverpool Inventory of Feelings and Experiences (O‐LIFE) (Mason, Linney, & Claridge, 2005). With a large body of evidence supporting shared genetic, environmental, neurobiological, and cognitive‐emotional factors between HS and psychotic disorders (Barrantes‐Vidal, Grant, & Kwapil, 2015; Ettinger, Meyhofer, Steffens, Wagner, & Koutsouleris, 2014; Kwapil & Barrantes‐Vidal, 2015; Nelson et al., 2013), the continuum model would predict that resting hippocampal perfusion is also elevated in individuals with subclinical psychotic‐like experiences. Although neuroimaging studies are yet to test this hypothesis, HS individuals show elevated hippocampal responses to a range of cognitive and emotional tasks (Bourque et al., 2017; Modinos et al., 2017b; Mohanty et al., 2005). Thus, our study sought to investigate whether hippocampal hyperperfusion is present in this group, compared to a matched group of people with low schizotypy. In addition, given that a recent study has shown associations between positive schizotypy scores and striatal functional connectivity at rest (including positive correlations between ventral striatum‐frontal cortex and negative correlations between dorsal striatum–posterior cingulate cortex) (Wang, Ettinger, Meindl, & Chan, 2018), and that vulnerability for psychosis has also been associated with hyperperfusion in dopamine signaling regions such as the striatum and the midbrain (Allen et al., 2017, 2016; Kindler et al., 2018), which could arise downstream from hippocampal hyperactivity (Lodge & Grace, 2011), we predicted that individuals with HS would also show increased resting perfusion in these subcortical regions.

## 2 MATERIALS AND METHODS

### 2.1 Participants

The study had King's College London Research Ethics Committee approval and all participants gave written informed consent to the study protocol. The study was conducted in compliance with the Code of Ethics of the World Medical Association (Declaration of Helsinki).

The recruitment procedure has been described in detail in our recent publications in a largely overlapping sample (Modinos et al., 2017a, 2017b). Briefly, 250 healthy individuals who responded to online advertisement were prescreened with the short version of the O‐LIFE questionnaire (Mason et al., 2005). Following previous imaging research in HS using the O‐LIFE (Premkumar et al., 2012), volunteers scoring high on the O‐LIFE Unusual Experiences (UE) subscale (UE > 7, *HS* group) and volunteers scoring low (UE < 2, *LS* group) were then invited to participate. The UE subscale of the O‐LIFE was used as it is associated with greater severity of positive symptoms in patients with psychosis (Cochrane, Petch, & Pickering, 2010), and because positive symptoms are the core criteria by which individuals at CHR of psychosis have been recruited to previous ASL studies (Allen et al., 2017, 2016). The O‐LIFE comprises three other subscales besides UE—including cognitive disorganization, impulsive nonconformity, and introvertive anhedonia—and a composite O‐LIFE total score may also be computed.

Twenty‐one individuals were included in the HS group (10 females; age range, 18–44 years; mean age 26.62 years) and 22 in the LS group (11 females; age range, 19–39 years; mean age 26.50 years). Participants were excluded if they had a personal history of neurologic/psychiatric disorders according to the Mini International Neuropsychiatric Inventory (Sheehan et al., 1998) as administered by a trained researcher. Other exclusion criteria included contraindications to MRI scanning, having a first‐degree relative with present/past history of psychotic disorder, present/past history of use of psychotropic medications, and use of recreational drugs in the two weeks prior to scanning or meeting criteria for substance abuse/dependency by self‐report.

### 2.2 Assessments

On the day of scanning, participants completed the following assessments: a validated short version of the Wechsler Adult Intelligence Scale‐III (WAIS‐III) to measure intelligence (Velthorst et al., 2013), a semi‐structured interview adapted from the Early Psychosis Prevention and Intervention Centre (EPPIC) Drug and Alcohol Assessment Schedule (http://www.eppic.org.au) to discard present/past use of antipsychotic medication, and to record present/past use of alcohol, tobacco, and cannabis; and the Social Functioning Questionnaire (SFQ) (Tyrer et al., 2005).

### 2.3 MRI acquisition

All scans were conducted on a General Electric Discovery MR750 3 Tesla system (General Electric, Chicago, IL) at the Institute of Psychiatry, Psychology and Neuroscience, King's College London using a 12‐channel head coil. A three‐dimensional T1‐weighted inversion recovery prepared gradient echo sequence, required for the preprocessing of the ASL images, was obtained (field of view: 270 mm, TR/TE/TI: 7.3/3.0/400 ms, slice thickness = 1.2 mm, 196 slices), based on the well‐validated ADNI 2/ADNI GO protocols (http://adni.loni.usc.edu/methods/documents/mri-protocols/).

A pseudo‐continuous ASL sequence was acquired with a multi‐shot, segmented 3D stack of axial spirals (8 arms) readout with a resultant spatial resolution of 2 × 2 × 3 mm. Four control‐label pairs were used to derive a perfusion‐weighted difference image (Dai, Garcia, de Bazelaire, & Alsop, 2008). The labeling RF pulse consisted of a train of one thousand Hanning‐shaped RF pulses with a total duration of 1.5 s and a postlabeling delay of 1.5 s was also used. This arterial blood RF labeling scheme has been shown to have a remarkably consistent labeling efficiency over a wide range of arterial blood velocities (Dai et al., 2008). The sequence included background suppression for optimum reduction of the static tissue signal, and used TE = 11.088 ms, TR = 4,923 ms, FoV = 240. Following the labeling of arterial blood, postlabeling delay and background suppression, the control and labeled images were both read with a “3D Fast Spin Echo Stack of Spiral” scheme (Thedens, Irarrazaval, Sachs, Meyer, & Nishimura, 1999). Within the whole‐brain excitation slab of the 3D scan, 56 “slice partitions” of 3 mm thickness were used. In the in‐plane direction, *k*‐space was sampled with a spiral raster consisting of 8 interleaved spiral arms of 512 points each. This spread of points has an equivalent voxel size (in‐plane) of 3.2 × 3.2 mm, *after* the spiral distribution of points is regridded into a rectangular matrix for Fourier Transformation. Once reconstructed, the image was written to a resulting rectangular matrix of 256 points in a 24 cm FoV, hence providing a final in‐plane voxel size of ∼2 × 2mm. As a result of the 180° pulses employed in the 3D “stack of spiral” Fast Spin Echo readout, any signal loss due to magnetic susceptibility gradients is refocused. Hence, our CBF images do not exhibit the well‐known signal dropout observed in other ASL methods that employ readout schemes such as Echo‐Planar Imaging. Within the same scanning series, a proton density image was acquired in 48 s using the same acquisition parameters to compute the CBF map (from the difference image) in standard physiological units (ml blood/100 g tissue/min). The whole ASL pulse sequence, including the acquisition of the proton density image, was performed in 6:08 min.

### 2.4 Preprocessing

CBF maps were preprocessed using FMRIB Software Library (FSL) software applications (http://www.fmrib.ox.a.c.uk/fsl) and Statistical Parametric Mapping (SPM12; http://www.fil.ion.ucl.ac.uk/spm/). Computation of CBF maps was performed using the following formula:

$$\text{CBF}=600\frac{e^{w/T_{1a}}}{2ɛT_{1a}(1-e^{-\tau /T_{1a}})}\frac{P}{\frac{R}{\lambda }}$$

in which *P* is the signal in the perfusion‐weighted image (control–label), *R* is the signal in the reference image, *ɛ* is the combined efficiency of labeling and background suppression (∼65%), τ is the labeling duration (∼1.5s), *T*_1a_ is the *T*_1_ of arterial water, and *w* is the postlabeling delay (∼1.5 s).

**Figure 1:**
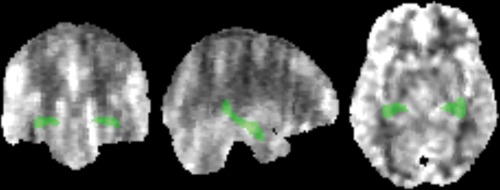
Representative subject‐level cerebral blood flow map in normalized space. In green, overlaid anatomical hippocampal mask from the Automated Anatomical Labeling atlas. The SNR in the hippocampus was measured to be ∼4.5 ± 1.0, as obtained from a set of 10 independent measures taken at random, by dividing the value of the perfusion induced signal over the noise and reporting the average. [Color figure can be viewed at http://wileyonlinelibrary.com]

**Figure 2:**
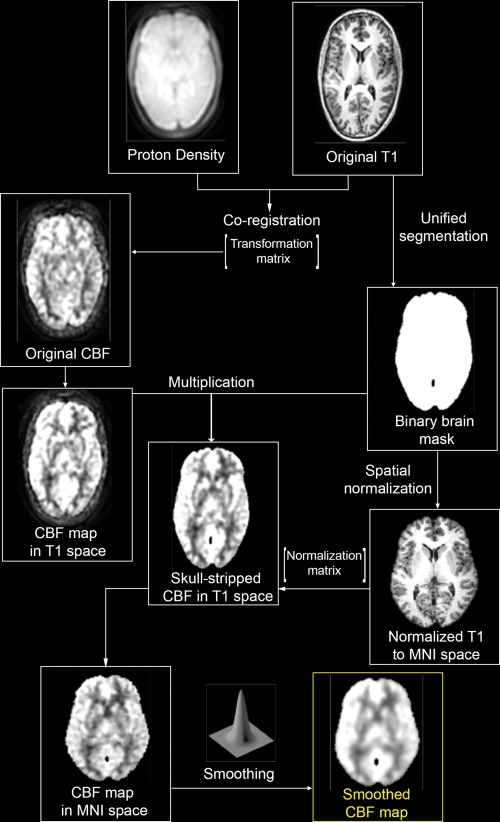
Arterial Spin Labeling preprocessing pipeline. [Color figure can be viewed at http://wileyonlinelibrary.com]

A multi‐step approach was performed for spatial normalization of the CBF maps to the space of the Montreal Neurological Institute (MNI): (a) co‐registration of the proton density image to the T1‐image after realigning the origin of both images. The transformation matrix of this co‐registration step was then applied to the CBF map, to transform the CBF map to the space of the T1‐image; (b) unified segmentation of the T1 image scan to generate a “brain‐only” binary mask; (c) elimination of extracerebral signal from the CBF map, by multiplication of the “brain only” binary mask above, with the CBF map in the space of the T1 image; (d) normalization of the subject's T1 and the skull‐stripped CBF map, using the parameters of the unified segmentation matrices. Finally, spatial smoothing of the normalized individual CBF maps (an example is shown in Figure 1) was carried out using a 6‐mm Gaussian smoothing kernel. A schematic view of the ASL preprocessing pipeline is shown in Figure 2.

### 2.5 Statistics

#### 2.5.1 Demographic data

Analyses of demographic data were performed in SPSS 24 (http://www-01.ibm.com/software/uk/analytics/spss/). The effect of group on these measures was examined using independent samples *t* tests for parametric data and Chi‐square tests for nonparametric data. Significant effects are reported at *p* < .05.

#### 2.5.2 Mean global CBF

To assess potential group differences in global CBF, global CBF values were extracted from each subject and an independent samples *t* test was performed in SPSS.

#### 2.5.3 ROI analysis

Statistical analyses of regional CBF data were implemented in SPM12. An independent samples *t* test was used to test for significant differences between the HS and LS groups using a voxel‐wise region of interest (ROI) approach. ROIs were specified *a priori* using the coordinates from a previous CBF study comparing CHR subjects with healthy controls (Allen et al., 2016): MNI coordinates in right *x*, *y*, *z* = 20, −28, −8 and left hippocampus *x*, *y*, *z* = −22, −28, −8; basal ganglia: right *x*, *y*, *z* = 22, −12, −4 and left *x*, *y*, *z* = −18, −8, −4 pallidum/putamen; and in the left midbrain *x*, *y*, *z* = −10, −32, −18. These coordinates were used for small volume correction (SVC) with a 6‐mm sphere. Statistical inferences were made at *p* < .05 with family‐wise error (FWE) correction for multiple comparisons at the voxel level after applying SVC. Mean global CBF was included as a covariate in the design matrix using ANCOVA, to account for interindividual differences in global perfusion.

Within the HS group, exploratory correlations between CBF in the hippocampal, basal ganglia and midbrain ROIs and severity of psychotic‐like experiences (O‐LIFE Total, UE, cognitive disorganization, impulsive nonconformity, and introvertive anhedonia scores) and with social functioning levels (SFQ scores) were tested with separate regression analyses in SPM, adjusted for mean global rCBF and using *p* < .05 with FWE correction at the voxel‐level after applying SVC.

Potential effects of age, gender, or substance use (alcohol, cigarettes, and cannabis) on the imaging findings were examined by including these variables as covariates of no interest in the SPM designs described above for group comparisons and correlations. Finally, for completeness, exploratory voxel‐wise whole brain effects were also assessed at a voxel‐wise *p* < .05 FWE threshold.

## 3 RESULTS

### 3.1 Demographic and questionnaire data

Table 1 summarizes the demographic and O‐LIFE questionnaire data in the HS and LS groups. By design, the groups only differed significantly in the schizotypy measure, with higher scores for the HS group in O‐LIFE Total (*p* < .001; Cohen *d* = 2.15), unusual experiences (*p* < .001; Cohen *d* = 3.61), cognitive disorganization (*p* < .001; Cohen *d* = 1.22), and introvertive anhedonia (*p* < .001; Cohen *d* = 1.51).

**Table 1:** Demographic and questionnaire data

Characteristic	Low schizotypy (*n* = 22)	High schizotypy (*n* = 21)	*t*/χ^2^	*p*
Age (years)	26.50 ± 5.13	26.62 ± 6.92	−.064	.949
Gender (male/female)	11/11	11/ 10	.024	.876
IQ (WAIS‐III short version)	122.45 ± 13.84	119.29 ± 16.73	.678	.502
O‐LIFE total	16.05 ± 8.78 [range: 4–33]	39.05 ± 12.32 [range: 24–58]	−7.022	<.001
O‐LIFE unusual experiences	1.00 ± 1.02 [range: 0–3]	12.14 ± 4.29 [range: 7–22]	−11.585	<.001
O‐LIFE cognitive disorganization	5.36 ± 4.02 [range: 0–12]	11.86 ± 6.34 [range: 5–24]	−3.989	<.001
O‐LIFE introvertive anhedonia	4.86 ± 3.23 [range: 1–14]	9.19 ± 2.46 [range: 5–13]	−4.926	<.001
O‐LIFE impulsive nonconformity	4.82 ± 4.34 [range: 0–15]	5.86 ± 4.26 [range: 0–17]	−.792	.433
Social Functioning Questionnaire total	4.14 ± 3.11	5.62 ± 2.88	−1.619	.113
Daily tobacco use	.74 ± 3.27	32 ± .77	.549	.586
Alcohol use (median [range])	2 (0–5)	1 (0–4)	5.017	.414
Marijuana use (median [range])	1 (0–3)	0 (0–3)	1.818	.611
Parental socio‐economic status (% salariat)	61.9% (*n* = 13)	73.7% (*n* = 14)	1.741	.419
Educational level (% postgraduate)	50% (*n* = 11)	38.1% (*n* = 8)	2.452	.653

*Note*. O‐LIFE = Oxford–Liverpool Inventory of Feelings and Experiences. WAIS‐III = Wechsler Adult Intelligence Scale‐III. Cannabis/alcohol use: 0 = never, 1 = experimental use (has tried occasionally), 2 = occasional use (has used small quantities from time to time), 3 = moderate use (has used in small quantities regularly/large amounts occasionally), 4 = severe use (has frequently used large quantities, often to intoxication/debilitation).

For the Social Functioning Questionnaire, higher scores indicate greater social impairment.

### 3.2 ASL data

#### 3.2.1 Mean global CBF

Group average mean global CBF values (ml/100 g/min) for the HS group were 48.88 ± 8.40, and for the LS group 49.30 ± 9.87, which did not differ significantly (*t*(41) = .152, *p* = .880; Cohen *d* = 0.05).

#### 3.2.2 ROI analysis

**Figure 3:**
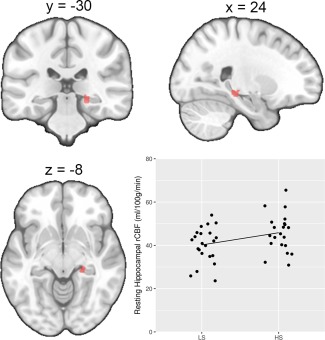
Increased resting perfusion in subjects with high positive schizotypy relative to subjects with low positive schizotypy in the right hippocampus (*p* = .031 FWE) and scatter plot showing hippocampal cerebral blood flow (CBF) levels in each group. [Color figure can be viewed at http://wileyonlinelibrary.com]

The *a priori* ROI analysis revealed higher CBF in the right hippocampus in subjects with HS compared to those with LS (MNI coordinates *x*, *y*, *z* = 24, −30, −8; *T* = 3.25; *Z* = 3.04; *k* = 42; *p* = .031 FWE‐corrected; Cohen *d* = 0.67) (Figure 3). There were no suprathreshold effects on the left hippocampus, basal ganglia, or midbrain regions. Exploratory voxel‐wise whole‐brain analysis did not reveal any significant differences.

Both the higher CBF levels in the HS compared to the LS group and the lack of further significant group differences remained apparent when age, gender, alcohol, cigarette, and cannabis use were added to the statistical model as covariates of no interest.

#### 3.2.3 Relationship between CBF and schizotypal or social functioning scores

No significant voxel‐wise correlations at *p* < .05 FWE were found between regional CBF and any of the O‐LIFE subscales or total score or SFQ scores.

## 4 DISCUSSION

The main finding of this study is that individuals with high positive schizotypy showed increased resting perfusion in the right hippocampus compared to those with low schizotypy. This finding was not attributable to group differences in global CBF levels. To our knowledge, this is the first application of the ASL method to investigate differences in resting perfusion in a schizotypy group. Given that the study groups only differed on the presence of schizotypal traits, and that hippocampal hyperperfusion has been reported in patients with psychosis (Friston et al., 1992; Liddle et al., 1992; Malaspina et al., 2004; Pinkham et al., 2011; Schobel et al., 2013, 2009; Talati et al., 2014, 2015; although see Andreasen et al., 1997; Catafau et al., 1994; Early et al., 1987; Parellada et al., 1994), and in people at CHR of sychosis (Allen et al., 2017, 2016; Schobel et al., 2013), our study findings suggest that increased hippocampal activity (reflected in an elevation of regional perfusion) is also involved in the expression of subclinical psychotic‐like experiences.

In terms of the clinical or functional correlates of CBF abnormalities along the psychosis spectrum, previous longitudinal studies in CHR individuals reported that (a) elevated hippocampal cerebral blood volume as measured with the contrast agent gandolinium was linked to the risk of later transition to psychosis (Schobel et al., 2013, 2009), (b) increased hippocampal CBF as measured with ASL was subsequently normalized in CHR subjects who remitted from their CHR state (Allen et al., 2016), and (c) hippocampal CBF and medial prefrontal GABA+ concentrations were associated in CHR individuals who subsequently developed a psychotic disorder (Modinos et al., 2018). Although the observation of an increase in resting hippocampal perfusion in HS is consistent with the majority of previous studies in CHR and schizophrenia with a specific focus on the hippocampus, as discussed above, the cross‐sectional nature of the present study prevents investigating whether this finding is prospectively linked to a subsequent emergence of mental health disorders in schizotypy (Cannon et al., 2015). Furthermore, although the significant effect on our sample was restricted to the right hippocampus, consistent with a recent study in CHR individuals compared to healthy controls (Allen et al., 2017), this observation is only partly consistent with other studies in CHR subjects in which hyperperfusion involved both hippocampi (Allen et al., 2016; Schobel et al., 2013). The lack of significant effects in the left hippocampus may be related to a lesser degree of psychosis risk, or to limited power in this nonclinical sample, and this will need to be replicated by independent studies of larger samples.

Contrary to our second hypothesis derived from CHR studies (Allen et al., 2017, 2016; Kindler et al., 2018), we did not find any group differences in resting perfusion of the basal ganglia or midbrain. Mechanistically, animal models strongly implicate hippocampal hyperactivity as a primary factor leading to downstream subcortical hyperdopaminergia and psychotic‐like behaviors (Lisman et al., 2008; Lodge & Grace, 2011). In contrast to CHR subjects, whilst individuals with HS endorse experiencing psychotic‐like phenomena of a subclinical nature, they are not help‐seeking, and have a lower likelihood of developing psychosis than a CHR group (Kwapil, Gross, Silvia, & Barrantes‐Vidal, 2013). As the hypothesis derived from animal models is that hippocampal hyperactivity is upstream from subcortical dopamine excess (Lodge & Grace, 2011), hippocampal hyperperfusion may be present to a lesser (although significant) extent in non‐help‐seeking HS subjects than in CHR individuals and patients with psychosis. As a consequence, the increase in mesolimbic dopaminergic regions may not be present or detectable in a sample of HS participants selected from a university population, and this could reflect mechanisms of resilience in this group. In CHR individuals, elevated striatal dopamine function is observed to a lesser extent than in psychosis (Howes et al., 2009), and this may further increase with transition to psychosis (Howes et al., 2011). Although there is some evidence of an association between altered dopaminergic neurotransmission and psychometrically identified schizotypy (Woodward et al., 2011), findings are less consistent than in frank psychosis, possibly due to high heterogeneity in the experimental designs and methods used (Mohr & Ettinger, 2014). Moreover, elevated stress‐induced dopamine release as measured with [^11^C]raclopride PET (Soliman et al., 2008) and increased functional activation as measured with functional MRI (Soliman et al., 2011) in striatal regions in schizotypy has only been observed in relation to negative schizotypal features (reflecting the interpersonal dimension of schizotypy, thought to mirror negative symptoms in schizophrenia), but not in relation to positive schizotypy (reflecting the cognitive‐perception dimension of schizotypy, thought to mirror positive symptoms in schizophrenia). Further research examining the relative contributions of hyperperfusion at different nodes of a hippocampal–striatal–midbrain circuit along a psychosis continuum (psychosis, CHR, and HS), combined with measurements of the different neurotransmitter systems involved (i.e., GABA, glutamate, and dopamine), may provide substantial insights into the neurobiology of risk and resilience for psychiatric disorders (Pantelis & Bartholomeusz, 2014). Considering that animal models have demonstrated a direct link between hippocampal excitation–inhibition imbalance and hippocampal hyperactivity (Lodge, Behrens, & Grace, 2009), and that GABAergic dysfunction is proposed as a key driver of hippocampal hyperactivity in schizophrenia (Heckers & Konradi, 2015), future preclinical studies could test whether selective suppression of hippocampal interneurons is related to increases in hippocampal CBF maps, and compare these to CBF maps in the extended human psychosis phenotype to dissect the molecular mechanisms underlying the dimensionality of psychotic experiences.

In terms of correlations with levels of psychopathology, this study did not find significant associations between severity of subclinical psychotic‐like experiences or levels of social functioning and resting perfusion in the hippocampus, basal ganglia, or midbrain. The two previous ASL studies in CHR subjects did also not observe significant correlations between levels of prodromal symptoms and hippocampal CBF (Allen et al., 2017, 2016). We cannot exclude the possibility that, by selecting subjects scoring above a certain threshold to determine high positive schizotypy, the range of O‐LIFE symptom scores was limited in both groups. Studies including a wider spectrum of individuals with schizotypy, subclinical, and overt psychosis may reveal relationships between hippocampal CBF and psychotic symptom severity.

There are some limitations to this study. First, subjects were recruited from a university sample, had relatively high IQs and groups showed no differences in self‐reported substance use; therefore, our results may not generalize to all individuals with schizotypy. Larger studies in samples drawn from the general population, and including biological measures of substance use such as a urine test, will help define the normal variation in schizotypy. Second, we selected individuals based on their scores on the unusual experiences subscale of the O‐LIFE and therefore the results reflect CBF changes associated with the positive dimension of schizotypy. Future research studying individuals on the basis of negative and disorganized schizotypal traits will be able to expand on the dimensional specificity of the present findings. Third, our sample was relatively small, and recruitment through self‐report has previously been related to higher rates in schizotypy scores (Linscott & van Os, 2010). Replication in larger, independent samples will be important to validate the current findings, perhaps including a measure of psychotic‐like experiences administered by a rater or clinician. Fourth, the distribution of schizotypy in nonclinical samples as measured with instruments such as the O‐LIFE or the Community Assessment of Psychic Experiences (CAPE) does not show a continuous normal distribution, but a continuous half‐normal distribution, with the majority of the population having very low values while a significant proportion has progressively higher values (van Os, Linscott, Myin‐Germeys, Delespaul, & Krabbendam, 2009). Furthermore, compared to other measures of schizotypy which are based on personality traits (e.g., Schizotypal Personality Questionnaire), the O‐LIFE includes more “pathological” items (e.g., does not tap on phenomena such as daydreaming), so that very low scores are “normal.” Thus, we used low scorers to reflect a normative group with absence of subclinical psychotic‐like experiences, although future studies including a control group based of average scorers should help expand these findings. Finally, in response to a reviewer recommendation, we tested for a potential relationship between hippocampal CBF and gray matter volume in this region. Individual CBF values extracted from the hippocampal region showing a significant group difference were introduced as covariate in an ANCOVA design in SPM12, using gray matter segments obtained after voxel‐based morphometry preprocessing as reported in a recent study by our group in this sample (Modinos et al., 2017a), following the proposal that GMV could represent a potential confounder in ASL studies (Asllani, Borogovac, & Brown, 2008; Chappell et al., 2011). This analysis yielded no significant effects within or between groups either at *p* < .05 FWE or at a more lenient threshold of *p* < .001 uncorrected. Thus, the observed group differences in hippocampal CBF in the present study are unlikely to be confounded by underlying GMV effects from this region. Noteworthy, our recent multimodal structural imaging study in this same sample did not find hippocampal volume differences between the groups (Modinos et al., 2017a), consistent with all other neuroanatomical investigations in schizotypy (DeRosse et al., 2015; Ettinger et al., 2012; Kuhn, Schubert, & Gallinat, 2012; Modinos et al., 2010; Nenadic et al., 2015; Wang et al., 2015; Wiebels, Waldie, Roberts, & Park, 2016).

## 5 CONCLUSIONS

Healthy subjects with psychometrically identified positive schizotypy show increases in resting perfusion of the hippocampal region as measured with ASL. These findings are consistent with preclinical and clinical evidence suggesting that increases in resting perfusion of the hippocampal region underlie the pathophysiology of high positive schizotypy, and indicate that such hyperactivity is not secondary to potential influences of disease chronicity or antipsychotic medication on brain function. Future studies specifically comparing individuals with different levels of psychotic‐like or clinically relevant psychotic symptoms over time could confirm whether increased hippocampal CBF represents a state or trait marker across the psychosis spectrum.

## CONFLICT OF INTEREST

GJB received honoraria for teaching from General Electric Healthcare, and acted as a consultant for IXICO, at the time of this study. The other authors declare no competing financial interests. Finally, we wish to thank George Gifford for permission to use his ASL and T1 scans as representative images for Figures 1 and 2 of this manuscript.
